# A service evaluation of the implementation of a novel digital intervention for hypertension self-monitoring and management system in primary care (SHIP): protocol for a mixed methods study

**DOI:** 10.1186/s12872-024-04279-y

**Published:** 2024-12-19

**Authors:** Anne Smith, Katherine L Tucker, Rebecca K Barnes, Cynthia Wright Drakesmith, Adaku Agwunobi, Paul A. Bateman, Anna Forbes, Simon de Lusignan, Gary A Ford, Takeshi Fujiwara, FD Richard Hobbs, Constantinos Koshiaris, Jonathan Mant, Brian McKinstry, Stephanie Pollock, Cathy Rice, Yaling Yang, Richard J. McManus

**Affiliations:** 1 Nuffield Department of Primary Care Health Sciences, Radcliffe Observatory Quarter, University of Oxford, Radcliffe Primary Care Building, Oxford, OX2 6GG UK; 2 Radcliffe Department of Medicine, Oxford University Hospitals NHS Foundation Trust, University of Oxford, Oxford, England; 3 Primary Care Unit, Department of Public Health and& Primary Care Research, University of Cambridge, Cambridge, England; 4 Centre for Medical Informatics, Usher Institute of Population Health Sciences and Informatics, University of Edinburgh, Edinburgh, Scotland; 5 Patient and Public Involvement contributor, Bristol, Scotland; 6 Brighton and Sussex Medical School, Brighton, UK

**Keywords:** Hypertension, Self-monitoring, Blood pressure, Telemonitoring, Digital intervention, Implementation

## Abstract

**Background:**

Hypertension is a key risk factor for death and disability, and blood pressure reduction is associated with significant reductions in cardiovascular risk. Large trials have shown that interventions including self-monitoring of blood pressure can reduce blood pressure but real-world data from wider implementation are lacking.

**Aim:**

The self-monitoring and management service evaluation in primary care (SHIP) study will evaluate a novel digital intervention for hypertension management and medication titration platform (“Hypertension-Plus”) that is currently undergoing initial implementation into primary care in several parts of the UK.

**Methods and analyses:**

The study will use a mixed methods approach including both quantitative analysis of anonymised electronic health record data and qualitative analyses of interview and customer support log data. Pseudonymised data will be extracted from electronic health records and outcomes compared between those using the digital intervention and their own historical data, as well as to those not registered to the system. The primary outcome will be difference in systolic blood pressure in the 12 months before and after implementation. A further analysis will utilise self-monitored blood pressure data from the Hypertension-Plus system itself. Semi-structured qualitative interviews will be completed with implementation and clinical leads, staff and patients in six general practices located in two different geographical areas in England. Informed by the non-adoption, abandonment, scale-up, spread, and sustainability (*NASSS*) framework, our analysis will identify the challenges to successful implementation and sustainability of the digital intervention in routine clinical practice and in patients’ homes.

**Ethics and dissemination:**

The analyses of pseudonymised data were assessed by the sponsor (The University of Oxford) as service evaluation not requiring individual consent and hence did not require ethical approval. Ethics approval for the qualitative analyses was provided by Wales REC 4 (21/WA/0280) and individual written informed consent will be gained for all participants. Results will be published in peer-reviewed journals, presented at national and international conferences and disseminated via patient and health service organisations.

**Discussion:**

This study will provide an in-depth analysis of the impact and acceptance of initial implementation of a novel digital intervention, enhancing our understanding and supporting more effective implementation of telemonitoring based hypertension management systems for blood pressure control in England.

## Background

Hypertension is a key risk factor globally for death and disability, and in the United Kingdom (UK) it is the most common long-term condition, currently recognised in over 8 million adults (14%) [1]. Improved blood pressure (BP) control is associated with significant reductions in cardiovascular risk: every 5mmHg systolic pressure reduction will reduce coronary heart disease (CHD) risk by approximately 10% and stroke risk by 20% [2].

However, such benefits are limited by factors such as health care system failure, clinical inertia in escalating BP lowering therapy and reduced patient adherence leading to sub-optimal use of proven interventions, notably antihypertensive medication [2, 3]. Key interventions which address these three issues, and have been shown to be cost-effective, include digital interventions facilitating patient self-monitoring and self-management [4, 5]. Both have been shown to support patient engagement and increased self-efficacy, to be effective at engaging clinicians, and to achieve improved BP control mainly through better management of medication. Little work has assessed the impact of such interventions on a wider scale. In Scotland, a simple text based telemonitoring system has been implemented and evaluated via the Scale-Up Programme [6]. To our knowledge, this will be the first evaluation in England of the “real-world” implementation into daily practice of a hypertension telemonitoring system, outside of the framework of a trial.

The self-monitoring and management service evaluation in primary care (SHIP) study aims to evaluate the challenges of implementing of a novel digital intervention in England and to assess its impact in terms of uptake, BP control and workload.

## Methods

### Study design and setting

The SHIP Study is a primary care based mixed methods study of the implementation of a digital self-monitoring/self-management intervention in general practices from three areas in England. The study comprises both quantitative and qualitative elements assessing real world implementation using routinely collected data alongside a qualitative process evaluation.

### Participants

Primary care practices implementing a novel digital intervention for hypertension management (the Hypertension-Plus service) will be invited to take part in this study by email approach initially followed by a remote site initiation meeting. The study will include pseudonymised data from people in participating practices including both users and non-users of Hypertension-Plus. To facilitate data extraction, practices need to be members of or prepared to join the Oxford Royal College of General Practitioners Clinical Informatics Digital Hub (ORCHID) [7].

### The intervention

The digital intervention (“Hypertension Plus”) was provided by Omron Healthcare combining blood pressure telemonitoring with prompts for hypertension management including medication titration intended to facilitate remote care [4]. The intervention is based on workflow algorithms developed from research data and decision support tools designed to be compliant with National Institute for Health and Care Excellence (NICE) guidelines [8].

The system combines a web-based dashboard for professionals, which both reads and writes data to the practice clinical system, with a mobile phone “app” which is downloaded by participating patients and used to upload BP readings. Patients are invited to use the digital intervention via contact from their practice and then sign up via a bespoke web interface. The choice of which patients receive an invitation is at the discretion of practices, but they are typically people with raised BP receiving antihypertensive treatment plus some without a diagnosis of hypertension. Telephone and online support is provided for those people having problems accessing or engaging with the technological platforms.

The digital intervention is a commercial system and is licenced annually, usually at local healthcare organisation level (known as primary care networks (PCN) or Integrated Care Boards in England). Implementation is being led by Omron working with the National Association for Primary Care (NAPC, https://napc.co.uk) and includes training for practices, support with identification of appropriate groups, support for patients, and peer led support facilitated by the NAPC.

### Quantitative study

Pseudonymised coded and numeric clinical data will be extracted using the Oxford-Royal College of General Practitioners Clinical Informatics Digital Hub (ORCHID) system [7]. ORCHID is a secure system that regularly extracts pseudonymised data from member practices for disease surveillance, quality improvement and research purposes (https://orchid.phc.ox.ac.uk/index.php/orchid-data/). Data will not be extracted from records of patients who have opted out of use of their health records in research. The analysis will take place within the ORCHID trusted research environment. Outside of ethically approved trials with individual consent, no individual data leaves this network. All aggregate data extracts subject to statistical disclosure control.

**Fig. 1:**
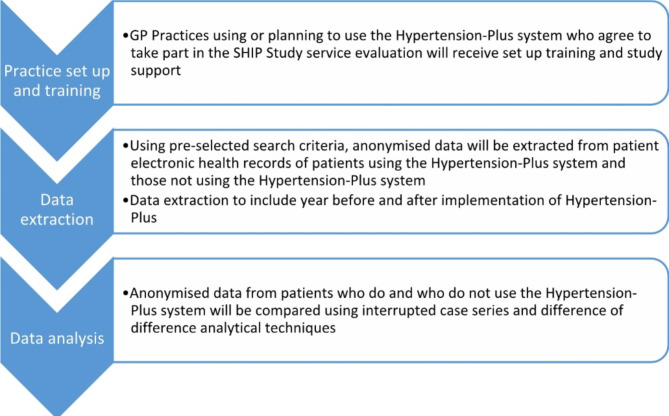
SHIP quantitative study procedures flow chart

People invited to use the digital intervention will be compared to patients on the practice hypertension register who have not been invited. Outcomes will be assessed using predefined curated variables of Systematised Nomenclature of Medicine (SNOMED) clinical terms (CT) codes (Fig. 1; see https://github.com/rmcmanusndpchs for codelists) [9].

(Fig. 1)

The most recent routinely collected retrospective data extracted at baseline will include:

- Age (years).
- Sex.
- Ethnicity.
- Smoking status.
- Index of Multiple Deprivation (IMD), a measure of socioeconomic status.
- Height, weight, body mass index (kg/m2).
- Total and HDL cholesterol (mmol/L).
- Systolic and diastolic blood pressure (clinic and/or home readings).
- Pulse.
- Past medical history:
  Diagnosis of diabetes (Type 1 or 2).
  Previous myocardial infarction or stroke.
  Diagnosis of chronic kidney disease.
- Medication use
  Statin treatment (drug type and dose).
  Antihypertensive medication use.
  Other current cardiovascular medication.
- Electronic Frailty Index (eFI) and/or characteristics required to calculate this.
- Health Economic Resource use: relevant consultations, referrals and admissions as available.
- Practice identifier (pseudonymised).

### Blood pressure data

Readings will be identified as having been taken by a healthcare professional in the clinic or by the patient at home. Where multiple readings are recorded on a single day, the lowest value will be selected. BP readings outside of the range systolic BP (SBP) < 70 or > 260 mm Hg or diastolic BP (DBP) < 40 or > 150 mm Hg will be discarded following standard editing rules [10].

### Data management

SNOMED CT code lists of the clinical features will be curated by clinical researchers, epidemiologists, and SQL developers to form the curated variables. The primary analysis will use Quality and Outcomes (QOF) business rules SNOMED clinical term definitions for the population and all relevant SNOMED codes to define co-morbidities [11]. QOF is a pay-for-performance chronic disease management system in the United Kingdom. Bespoke algorithms will be developed for data extraction, filtration, manipulation, and exploratory data analysis (EDA). Code lists are available (https://github.com/rmcmanusndpchs).

### De-identified intervention data

In addition to the routine clinical data extracted via ORCHID from the electronic health record, anonymised ‘process’ measures (including how many registered, medication, co-morbidity and self-monitored BP data) will be separately extracted from the stand-alone Hypertension-Plus database. Further information regarding this is presented after the ORCHID analysis.

### Outcomes

### Primary outcome

The primary outcome will be change in systolic blood pressure (SBP, clinic and home BP both included) in mmHg extracted from the electronic healthcare record comparing 12 months before and after intervention implementation along with comparison between those on the hypertension register invited to use and not invited to use the intervention.

### Secondary outcomes

Diastolic blood pressure (DBP) in mm Hg, extracted from the electronic healthcare record or the Hypertension-Plus system in the same manner as SBP readings using the same comparison as the primary outcome. A sensitivity analysis considering SBP and DBP in the same way as the primary analysis will include those with uncontrolled BP prior to implementation of the intervention. Controlled BP will be defined as SBP < 140 mm Hg and DBP < 90 mm Hg for clinic readings or SBP < 135 mm Hg and DBP < 85 mm Hg for home readings. Uncontrolled BP will be defined as the presence of any clinic or home SBP or DBP reading at or above these thresholds.

Primary care hypertension workload defined as the number of consultations where hypertension or hypertension monitoring was listed as the primary problem will be compared before and after implementation in a similar way to the BP outcomes.

### Exploratory outcomes

Exploratory outcomes include the evaluation of the impact of the digital intervention on antihypertensive medication use and other cardiovascular preventative treatment (statins, antiplatelets, anticoagulants). Subgroups (see below) will be examined for the comparisons defined in the primary outcome.

Further exploratory outcomes will be addressed using the standalone intervention data. These are detailed below in the Hypertension-Plus outcomes and analysis section.

### Sample size

Sample size requirements for this study were determined through simulation. A mean BP of 148 mm Hg was assumed, with practice variation (standard deviation, SD) of 5 mm Hg. Within a practice, patient variation (SD) was assumed as 17 mm Hg and measurements within a patient were assumed to vary by 10 mm Hg (SD) about the patient mean. Effect size was assumed to be -3 mm Hg (BP 3 mm Hg lower in patients using the digital intervention) and a minimum of 4 BP measurements for each patient. This demonstrated that to achieve 90% power at the 5% significance level, 100 patients each drawn from 8 general practices would be required for this study (800 patients in total, 91% power). Assuming greater variation (practice SD = 10 mm Hg, patient SD = 22 mm Hg or measurement SD = 14 mm Hg), the required sample size was 100 patients from 14 practices respectively. The available sample will depend upon the implementation rate as well as recruitment and will include as many patient records as are available.

### Statistical analysis

Descriptive statistics will be presented as the means and standard deviation (SD), proportions, and medians with interquartile ranges where appropriate. In the efficacy analyses for blood pressure, mixed-effects model repeated measures will be used to compare the changes in BP for people on the hypertension registers (i.e. with a clinical code for hypertension) between one year before and one year after implementation of the digital intervention within each practice. Fixed effects in the model are the exposure, whether BPs are measured (home or clinic), periods (12 months before and after implementation), interaction terms between the exposure and time period variable and covariates that were selected a priori because they have been reported to show correlations with both BP and cardiovascular (CVD) risk and could potentially confound the association between BP and CVD risk. These potential confounders will include baseline demographic characteristics (age, sex, ethnicity, IMD and frailty), clinical characteristics at baseline (SBP and DBP, total cholesterol, smoking, antihypertensive medication use), past history (diabetes, established cardiovascular disease, and chronic kidney disease). Random effects for patient and practice level average BP will also be included in the model. These covariates were selected a priori because they have been reported to show correlations with both BP and CVD risk and could potentially confound the association between BP and CVD risk [12]. Covariates are defined using the closest available electronic healthcare records data prior to baseline. The intervention analysis will take a similar approach albeit limited to the self-monitoring group and the variables included in the Hypertension-Plus dataset.

A 5% significance level will be used for all analyses/ tests.

### Subgroups

Analyses and modelling above will be repeated in the following subgroups: (BOX 2)

- Male vs. female.
- Previous history of cardiovascular disease defined here as myocardial infarction or stroke/TIA.
- People with baseline (clinic) blood pressure ≤ 140/90 mmHg compared to those > 140/90mmHg.
- Deprivation score (High vs. Low) worst 3 quintiles vs. best 2 quintiles.
- Ethnicity (white vs. other ethnicity).
- Geographical Area comparing the three areas where the system has been implemented.

### Cost analysis

If possible, the costs of National Health Service (NHS) primary care hypertension management including both medical consultations and antihypertensive prescriptions between the patient groups with and without the implementation of the digital intervention will be compared before and after the implementation period. The impact on NHS cost from implementation of Hypertension-Plus will be examined by comparing both before and after and between the intervention and the no-intervention groups. Data are likely to be limited due to inconsistencies in practice and practitioner level recording of activity and a decision will be made following data extraction if sufficient data are available to undertake these analyses.

### Digital Intervention data outcomes and analysis

#### Digital intervention outcomes

For the data extracted directly from Hypertension-Plus, the outcomes of interest will be:

1) the mean change in home SBP level comparing baseline BP (first week of measurement) with BP in (or next available after) the 6th month of measurement.

2) the number/proportion of people who initially onboarded that subsequently measure their BP and transmit it to the system at least once and time (days) between onboarding and first recorded measurement.

3) the mean change in home DBP level from baseline after the 6 months of home BP telemonitoring, comparing baseline BP with BP in (or next available after) the 6th month of measurement.

Depending on data availability we will do more exploratory analyses on:

Subgroups: outcomes 1–3 will be assessed in 10-year age intervals.

### Statistical analysis of Digital Intervention data

Baseline BP will be defined as the mean BP of the first BP measurement cycle. BP at 6 months will be defined as the mean BP value corresponding to the cycle 6 months after the first measurement cycle (or the next available data after 6 months if data after 6 months is not available). In the efficacy analyses for BP, mixed-effects model repeated measures will be used to compare the changes in BP from baseline to 6 months. Baseline patients’ characteristic will be set as fixed effects and registered GP practice and patient as random effects in this model. Baseline patients’ characteristics include demographic variables (age, gender, ethnicity, frailty and multimorbidity), past history (type 2 diabetes, chronic kidney disease or type 1 diabetes, renal disease, heart failure, cardiovascular (CVD) event, increased CVD risk [estimated 10 years], and postural BP drops) and antihypertensive medication use.

### Qualitative study

Guided by the ‘Non-adoption, Abandonment, and Challenges to the Scale-Up, Spread, and Sustainability’ (NASSS) framework [13], qualitative methods will be used to assess challenges to the successful (or otherwise) implementation and sustainability of Hypertension-Plus. We will collect and analyse qualitative data via semi-structured interviews with implementation and clinical leads, and staff and patients associated with GP surgeries adopting the intervention, as well as anonymised OMRON customer support data logs.

### Data collection

#### Patient interviews

Semi-structured qualitative interviews will be held with approximately 20 patients as adopters of the intervention recruited via practice mail outs. The sample will include patients who were unwilling to adopt the intervention, patients who were willing but unable to maintain use, and patients who adopted the intervention and sustained their usage. Interviews will be used to understand patients’ experiences and views of the intervention. Interviews will be conducted by telephone or remote video and audio-recorded. Informed consent will be sought by the researcher before the start of all interviews.

### Healthcare professional interviews

Semi-structured qualitative interviews will be held with approximately 10–15 health care professionals (e.g., General Practitioners (GPs), nurses, pharmacists), representing all the study practices, as adopters involved in implementing the intervention in GP practices. Interviews will be conducted by telephone or remote video. Following informed consent, the researcher undertaking the interviews will invite health care professionals to reflect on their experiences of implementing the intervention, factors predicting or influencing intervention adoption, implementation and maintenance, and their thoughts on the impact of self-monitoring of BP on the target population.

### Implementer / clinical lead interviews

Semi-structured qualitative interviews will be held with approximately 15 implementation leads involved in rolling out Hypertension-Plus in two geographical areas and clinical leads from each of the participating GP surgeries. Leads will already have been identified at GP surgery / Primary Care Networks and via the National Association for Primary Care as part of the first phase roll out plan and will be invited by the study team. Interviews will be conducted by telephone or remote video. Following informed consent, the researcher undertaking the interviews will invite leaders to reflect on the wider context, their experiences of implementing the intervention at the organisational level (including their strategy and priorities for roll-out), any feedback from staff and their long-term view of sustainability.

### Omron customer support data logs

Records of customer support data in the form of anonymised support forms/email enquiries from Omron will be collected to gain insight into technological factors predicting or influencing intervention implementation and maintenance.

### Qualitative data analysis

Recordings, transcripts and customer support data will be uploaded to NVivo and analysed by members of the research team using the Framework Method [14]. The Framework Method has five stages: familiarisation; identifying a framework; indexing; charting; and mapping and interpretation. After listening to the recordings, reading transcripts and other written data and discussing impressions, thoughts and ideas in light of the research objectives, preliminary codes will be applied highlighting different aspects of the participants’ experiences, against illustrative extracts from the data. Framework categories will then be developed for the purpose of sifting and sorting the datasets informed by the seven domains in the NASSS framework to structure observations concerning factors predicting or influencing intervention adoption, implementation and maintenance, but also incorporating any additional issues pertinent to participants derived from the data. After piloting and refining the framework on a subset of data, the categories will be used to index each note and transcript in NVivo 12. The indexed data for each framework category will then be summarised and organised by the framework categories in chart form to facilitate within and between-case analyses to enable the mapping and interpretation of findings in light of the research question.

### Sponsor and permissions

The SHIP Study is sponsored by the University of Oxford, Clinical Trials and Research Governance, Joint Research Office, Block 60, Churchill Hospital, University of Oxford, Oxford, OX3 7LE.

Ethics approval for the qualitative aspects for the qualitative aspects of the study was provided by Wales REC 4 (21/WA/0280). The quantitative cohort analyses were assessed by the sponsor as a service evaluation for which ethical permission was not required.

### Dissemination

Research outputs from this work will be published in peer-reviewed journals, presented at scientific conferences and via lay and social media (e.g., Twitter, blogs). ‘Patient friendly’ study summary documents and infographics will be made available to all participants at the end of the study via the study website and we will work with relevant patient groups (e.g. Blood Pressure UK) and health organisations (e.g. the primary care networks) to support dissemination to relevant groups and wider public.

## Discussion and conclusion

This mixed methods service evaluation aims to provide a greater understanding of the impact of initial implementation of the Hypertension-Plus digital intervention. It will allow comparison with the results from contemporary hypertension self-monitoring trials.

The results of this work will enhance understanding of self-monitoring of BP and enable better targeted and more effective implementation of SMBP. The qualitative process evaluation will give insight into the challenges to the successful (or otherwise) implementation and sustainability of the system.

## Data Availability

Quantitative data for this study are not available as access for the current analysis has been provided by agreements which preclude provision to third parties. Qualitative data are available from the authors upon reasonable request and with permission of the University of Oxford. Use of data will require approval by an independent review committee identified for this purpose and investigators requesting data will need to provide a written protocol including analysis plan and sign a data sharing/access agreement. Requests for Data Sharing should be directed to information.guardian@phc.ox.ac.uk.

## Abbreviations

BP: Blood pressure
CHD: Coronary heart disease
DBP: Diastolic BP
Efi: Electronic Frailty Index
EDA: Exploratory data analysis
GP: General Practitioner
HDL: High Density Lipoprotein
IMD: Index of Multiple Deprivation
NAPC: National Association for Primary Care
NHS: National Health Service
NICE: National Institute for Health and Care Excellence
NASSS: Non-adoption, abandonment, scale-up, spread, and sustainability
ORCHID: Oxford Royal College of General Practitioners Clinical Informatics Digital Hub
PCN: Primary care networks
QOF: Quality and Outcomes
REC: Research Ethics Committee
SQL: Structured query language
SD: Standard deviation
SNOMED CT: Systematised Nomenclature of Medicine clinical terms
SBP: Systolic BP

## References

[1] Lim SS et al. A comparative risk assessment of burden of disease and injury attributable to 67 risk factors and risk factor clusters in 21 regions, 1990–2010: a systematic analysis for the global burden of Disease Study 2010. Lancet, 2013. 380. 10.1016/S0140-6736(12)61766-8 PMC4156511 23245609

[2] Law MR, Morris JK, Wald NJ. Use of blood pressure lowering drugs in the prevention of cardiovascular disease: meta-analysis of 147 randomised trials in the context of expectations from prospective epidemiological studies. BMJ. 2009;338:b1665. 19454737 10.1136/bmj.b1665 PMC2684577

[3] Ogedegbe G. Barriers to optimal hypertension control. J Clin Hypertens (Greenwich). 2008;10(8):644–6. 18772648 10.1111/j.1751-7176.2008.08329.x PMC8109930

[4] McManus RJ, et al. Efficacy of self-monitored blood pressure, with or without telemonitoring, for titration of antihypertensive medication (TASMINH4): an unmasked randomised controlled trial. Lancet. 2018;391(10124):949–59. 29499873 10.1016/S0140-6736(18)30309-X PMC5854463

[5] Tucker KL, et al. Self-monitoring of blood pressure in hypertension: a systematic review and individual patient data meta-analysis. PLoS Med. 2017;14(9):e1002389. 28926573 10.1371/journal.pmed.1002389 PMC5604965

[6] Hammersley V, et al. Telemonitoring at scale for hypertension in primary care: an implementation study. PLoS Med. 2020;17(6):e1003124. 32555625 10.1371/journal.pmed.1003124 PMC7299318

[7] de Lusignan S, et al. The Oxford Royal College of General Practitioners Clinical Informatics Digital Hub: protocol to develop extended COVID-19 surveillance and trial platforms. JMIR Public Health Surveill. 2020;6(3):e19773. 32484782 10.2196/19773 PMC7333793

[8] Nationl Instititute for Health and, Excellence C. Hypertension in Adults: diagnosis and management - NICE. 2019; https://www.nice.org.uk/guidance/NG136

[9] de Lusignan S. Codes, classifications, terminologies and nomenclatures: definition, development and application in practice. Inf Prim Care. 2005;13(1):65–70. 10.14236/jhi.v13i1.580 15949178

[10] Stergiou GS, et al. White coat effect detected using self-monitoring of blood pressure at home: comparison with ambulatory blood pressure. Am J Hypertens. 1998;11(7):820–7. 9683043 10.1016/s0895-7061(98)00038-7

[11] England N. Quality and Outcomes Framework (QOF) business rules v47.0 2022–2023 baseline release. 2023, NHS England: London.

[12] Williams B, Spiering MG, Agabiti Rosei W, Azizi E, Burnier M, Clement M, Coca DL, de Simone A, Dominiczak G, Kahan A, Mahfoud T, Redon F, Ruilope J, Zanchetti L, Kerins A, Kjeldsen M, Kreutz SE, Laurent R, Lip S, McManus GYH, Narkiewicz R, Ruschitzka K, Schmieder F, Shlyakhto RE, Tsioufis E, Aboyans C, Desormais V, ESC Scientific Document Group. 2018 ESC/ESH guidelines for the management of arterial hypertension. Eur Heart J. 2018;39(33):3021–104. 30165516 10.1093/eurheartj/ehy339

[13] Greenhalgh T, et al. Beyond adoption: a New Framework for Theorizing and evaluating nonadoption, abandonment, and challenges to the Scale-Up, Spread, and sustainability of Health and Care technologies. J Med Internet Res. 2017;19(11):e367. 29092808 10.2196/jmir.8775 PMC5688245

[14] Ritchie J. S.L., Qualitative data analysis for applied policy research. In A. Bryman and R.G.Burgess, editors Analysing qualitative data, (pp.173–194). Routledge, London, 1994.

